# HistoEnder: A 3D printer-based histological slide autostainer that retains 3D printer functions

**DOI:** 10.1016/j.ohx.2022.e00370

**Published:** 2022-10-23

**Authors:** Marco Ponzetti, Ganga Chinna Rao Devarapu, Nadia Rucci, Armando Carlone, Vittorio Saggiomo

**Affiliations:** a Department of Biotechnological and Applied Clinical Sciences, University of L’Aquila, Via Vetoio, L’Aquila 67100, Italy; b Centre for Advanced Photonics & Process Analysis, Munster Technological University, Rossa Avenue, Bishopstown, Cork T12 P928, Ireland; c Tyndall National Institute, Lee Maltings, Prospect Row, Cork T12R5CP, Ireland; d Department of Physical and Chemical Sciences, Università degli Studi dell’Aquila, Via Vetoio, L’Aquila 67100, Italy; e Laboratory of BioNanoTechnology, Wageningen University and Research, Bornse Weilanden 9, Wageningen, The Netherlands

**Keywords:** Slide autostainer, Histology, Pathology, HistoEnder, Haematoxylin Eosin, Dip-coating

## Abstract

Automated microscope slide stainers are usually very expensive and unless the laboratory performs heavy histological work it is difficult to justify buying a 2000-10000€ machine. As a result, histology and pathology labs around the world lose thousands of working hours for following procedures that could be easily automated. Herein, we propose a simple modification of an open-source 3D printer, the Creality Ender-3, into an automated microscope slide autostainer, the HistoEnder. The HistoEnder is cheap (less than 200€), modular, and easy to set up, with only two 3D-printed parts needed. Additionally, the 3D printer retains its full functionality, and it can be reverted back into 3D printing in less than 1 min. The g-code associated with the procedure is extremely simple, and can be written by anyone. The HistoEnder can also be used in chemistry and material science laboratories for automating surface modifications and dip coating.

## Graphical abstract

**Figure:**
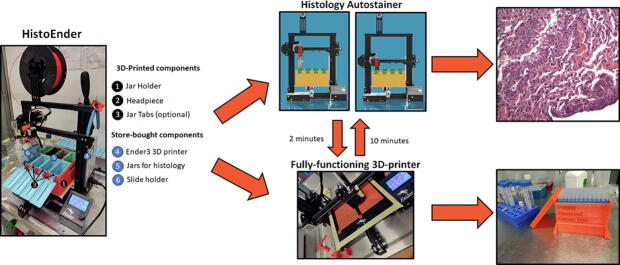

## Specifications table

**Table:** 

Hardware name	HistoEnder
Subject area	• Histology • Pathology • Biological Sciences • Material Sciences
Hardware type	Microscope slides autostainer
Closest commercial analog	Microscope slides autostainer
Open source license	CC-BY 4.0
Cost of hardware	€190
Source file repository	Live version on GitHub: https://github.com/Ponz91/HistoEnder repository on Zenodo: https://zenodo.org/badge/latestdoi/537764424https://doi.org/10.5281/zenodo.7088610

## Hardware in context

Histological and histopathological techniques are the bases upon which much of our current knowledge of humans, animals and plants is founded [1]. These procedures generally require investigators to collect a sample (e.g. an organ or a biopsy), process it so that it can be embedded in a support medium [paraffin, polymethylmethacrylate (PMMA), optimal cutting temperature (OCT) medium], cut in 3–30 μm-thick slices, and then stained to visualize the otherwise visible-light-transparent cellular structures of the tissue [2]. The staining requires the collection of the slices onto glass slides to facilitate handling, and then the removal of the support medium, followed by the hydration of the tissue section. This is achieved by first passing the slide-mounted slices through a medium-specific solvent (xylene or xylene-substitutes for paraffin, methoxyethyl acetate for PMMA, water for OCT), a transition solvent (usually ethanol or ethanol/isopropanol), and then descending concentrations of transition solvent in water. Eventually, when the tissue is in aqueous medium, the staining can occur. After the staining, slides have to be protected to preserve the best tissue morphology possible, and this is done by applying a thin glass slide, named coverslip, on top of the tissue (mounting). To avoid damage to the tissue, a mounting medium is applied between the slide and the coverslip. The mounting medium is often xylene-soluble, therefore slides have to be dehydrated with ethanol and then put in xylene before mounting. After curing, the slides can finally be observed with a brightfield microscope [2]. Needless to say, this procedure is time-consuming when performed manually, and even the simplest staining can take investigators hours of hands-on time, since passages from one step to the next are not immediate, and there is always an incubation time in the various solutions through which the slides need to go. A possible solution would be automating the procedure, which is conceptually quite easy to conceive. However, commercially available autostainers cost between 2000€ and 10000€. A cheap, easy to use, compact, and modular machine for automatic slide staining is still lacking. Fused Deposition Modeling (FDM) 3D printers, because of their simplicity and low cost, have been already modified to produce syringe pumps [3], bioprinters [4], [5] and in-incubator microscopes that allow for live cell monitoring and analysis [6]. In this report we fill the gap and modify a cheap 3D printer to build an automatic slide stainer, and we call this machine HistoEnder.

The HistoEnder is an open-hardware project based on a Creality Ender-3 open-source 3D printer [7], which allows users to automatically stain tissues mounted on microscope slides. The HistoEnder is also versatile and can perform dip coatings [8] automatically with minimal non-permanent modification of the printer. The HistoEnder will make automatic stainings accessible even to low-budget laboratories, laboratories that only perform staining occasionally and cannot justify the cost of an autostainer, and laboratories that want to own a 3D printer because of its versatility as a tool and also perform histological stainings/dip coatings, and more.

## Hardware description

**Fig. 1:**
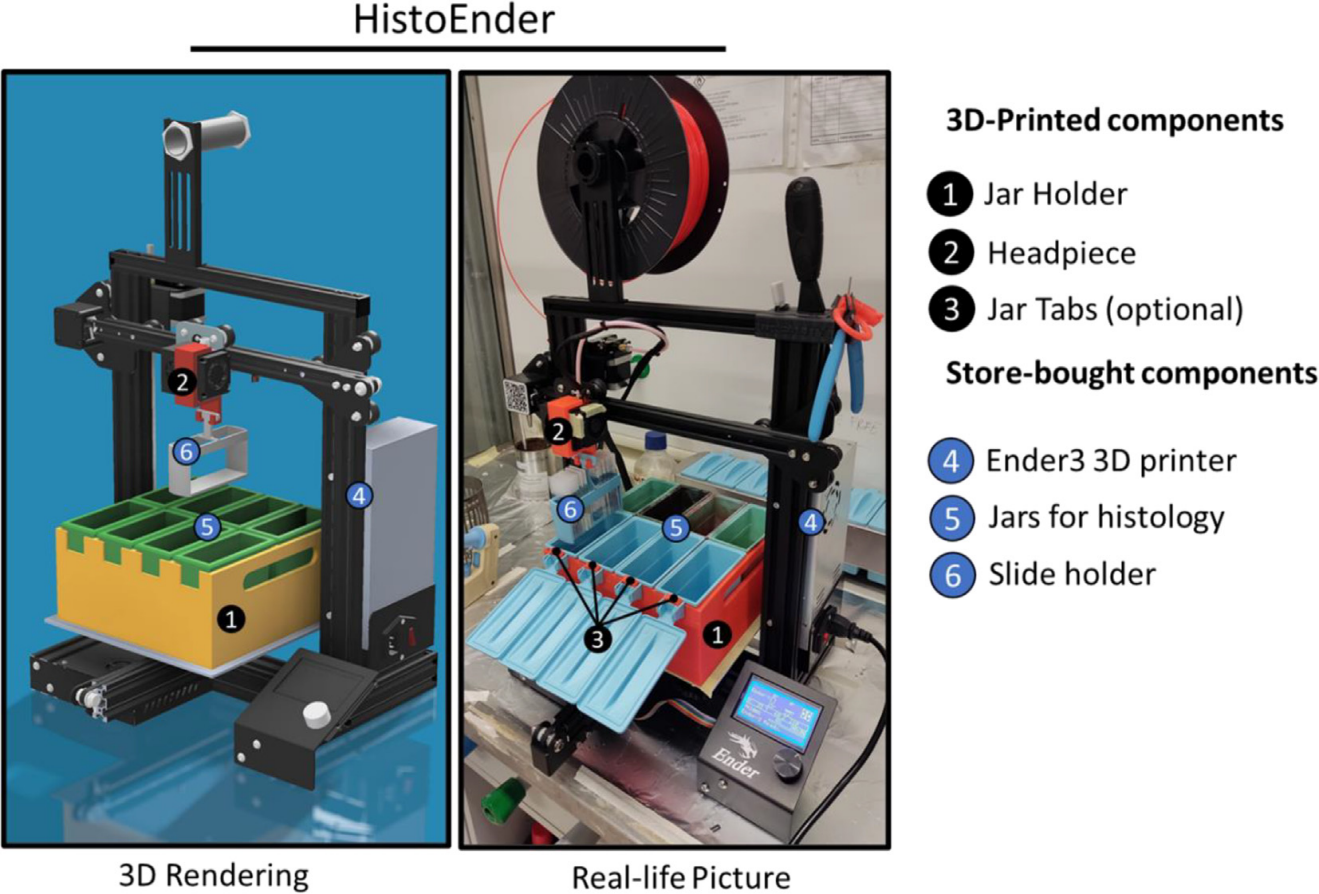
**3D rendering and real-life picture of the HistoEnder system.** Numbers indicate parts specified in the legend.

The HistoEnder (Fig. 1) is based on a Cartesian FDM 3D printer, and works by moving a slide rack loaded with microscope slides through different solvent/staining jars, in a fully programmable fashion. This makes the machine incredibly versatile and able to perform several cytological/histological staining jobs automatically.

The 3D printer is modified just by adding two additional components: a jar holder for keeping the staining jars on the printer bed, and a headpiece for mounting the slide holder. Additional 3D-printed pieces such as an alignment help are suggested and described in this manuscript, but not strictly required. Importantly, the 3D printer retains full printing capabilities which can be restored in less than one minute by removing the additional 3D printed parts. The HistoEnder is easy to use, and after an initial set-up and a dry run test can be used right away with minimal run-to-run adjustment, thanks to the precise homing mechanism of the 3D printer itself. Programs are easy to modify even with minimal knowledge of g-code, and we are currently working on a web application which will also automate this process. Replacing the headpiece with a clamp holder allows this machine to perform surface modification and dip coating at different speeds, making the HistoEnder useful for material scientists and chemists as well. The only real limitations being the size of the bed, which allows for eight 25-slides staining jars to be used, and the low movement speed of the Z axis (dip-raise motion) which has to be accounted for when performing fine-tuning of staining time.

In summary, the HistoEnder:

• Saves hours of manual staining to researchers who currently do such procedures by hand
• Increases staining consistency between runs thus increasing experimental reproducibility
• Can be adapted to a variety of procedures starting from smears, paraffin-embedded sections, OCT-embedded cryosections and even PMMA-embedded hard tissue samples
• Can perform dip coatings for material science and chemistry laboratories

### Design files

*All STL files and g-codes are available with the article and on the github repository of the project* [9] *.*

## Design files summary

**Table:** 

Design file name	File type	Open source license	Location of the file
Jar holder	F3D, STL	CC-BY 4.0	GitHub/Zenodo
Headpiece	F3D, STL	CC-BY 4.0	GitHub/Zenodo
Jar tabs	F3D, STL	CC-BY 4.0	GitHub/Zenodo
Clamp Holder	STL	CC-BY 4.0	GitHub/Zenodo
Alignment guide	F3D, STL	CC-BY 4.0	GitHub/Zenodo
Setup guide	MP4	CC-BY 4.0	Available with the article and on YouTube [10]

## Parts description

### Jar holder

This is the 3D-printable holder piece that will keep all the staining jars in their correct positions onto the printer bed. The cutouts for the jars are 46.4 mm by 102.5 mm, and are spaced 6 mm between one another to allow for the 2.8 mm overhang of the jars. The holder is 100 mm in height.

### Headpiece

The headpiece is press-fit onto the printhead and has a hook to hold several of the most common models of slide rack and keeps it aligned with the nozzle so that the XY offset is negligible.

### Jar tabs

Jar Tabs are only needed by investigators who want to use the HistoEnder with their embedded-lid staining jars. These tabs keep the lids from colliding with the Y motor assembly and belt system of the printer.

### Clamp holder

A clamp which can be magnetically attached on the printhead using a 1x1x1 cm neodymium magnet. The clamp can hold a single microscope slide, paper, and other materials to be dip-coated, for example in different beakers.

### Alignment guide

An alignment guide is optional, but strongly suggested. It’s a simple jig that sits onto the left bottom corner of the 3D printer bed, and guides the user into placing the Jar holder onto the bed precisely.

### Setup guide

A step-by-step video guide to setting up the HistoEnder after all printed parts are ready.

### Bill of materials

The project’s bill of materials is short, and the only parts that the researcher needs are the 3D printer, the staining jars, and a spool of Polyethylene terephthalate glycol-modified (PETG). The files are designed to fit one of the most common jar sizes available on the market (∼45.6 × 101.3 × h93.4 mm without lid), therefore most histologists and pathologists will only need to buy the 3D printer, and PETG, making the project even more affordable.

## Bill of materials summary

**Table:** 

Designator	Component	Number	Cost per unit -currency	Total cost - currency	Source of materials	Material type
3D-printed parts	1	1	€14	€14	3DJake [11] (spool)	PETG
Staining Jar and slide holder	5	8	€3.2	€25.6	Alibaba [12]	Polyoxymethylene
Creality Ender-3	6	1	€150	€150	Alibaba [13]	Non-specific
10x10x10mm Magnet (for clamp holder)	1	1	€0.66	€0.66	Aliexpress [14]	NdFeB
Masking tape	7	4 strips	€0.025	€0.01	Hardware store	Paper
Hairspray or glue stick	–	to cover the printer bed	€0.01	€0.01	Hardware or Stationery store	Non-specific

All the 3D printed parts can be printed out of half a single spool of PETG (approx. 450 g of raw material is needed).

## Build instructions

If the researcher does not already have an Ender-3 3D printer:

1. Build the Ender-3 as described in the instruction manual of the printer. The system is set to work with the supplied bed (rubber mat style), but it will also work with a glass bed or a magnetic bed. However, the g-code needs to be modified to account for the difference in the height of the bed.

Printing the parts:

2. Attach the print bed sheet to the heating bed using masking tape on the sides. Do not use the clips that come with the printer because that will result in a reduction in the printable area on the bed.
3. Carefully level the bed and run a bed leveling test print; can be done automatically with CR-touch sensor as well for easier alignment. It is crucial that the bed is perfectly leveled since the Jar Holder print uses the entire bed area. Also carefully calibrate the printer so that XYZ and E are accurate. Plenty of online tutorials are available for those who are new to 3D printing (some of YouTube channels with tutorials are Thomas Sanladerer, CNC Kitchen, Teaching Tech, Maker's Muse and many others).
4. Print all parts in PETG. Settings used in Ultimaker Cura 4.11.0:
  a. Nozzle size: 0.4 mm.
  b. Walls: 2.
  c. Top/bottom layers:4.
  d. Nozzle temperature: 250 °C or follow manufacturer’s indications.
  e. Bed temperature: 80 °C (to minimise warping) or follow manufacturer’s indications.
  f. Fan speed: 0 % (to maximise layer adhesion).
  g. Print speed: 50 mm/s.
  h. First layer print speed: 25 mm/s.
  i. Infil: 30 % tri-hexagon.
  j. Retraction: 2 mm.
  k. Support: Normal; everywhere.
  l. Build plate adhesion: none.
  m. Hairspray was used to create an adhesive surface on the bed, glue stick would work just as well. This point can be avoided with adequate settings and levelling.
5. Carefully remove supports from the 3D printed parts. Users might choose to use tree supports for the headpiece instead of normal supports for easier removal.
• Fitting the HistoEnder with its custom pieces:
6. Set 8 empty staining Jars in the Jar Holder, with their lid off/open.
  a. If a jar model with an embedded lid is to be used, the researcher must use the tabs to keep the lids open. Alternatively, they can be unhinged (at the users’ own risk).
7. Raise the Z axis using the controls on the printer to a comfortable height and move the X axis towards the middle of the bed using the printer control system, and test-fit the head piece. The triangular notch should align with the nozzle (Fig. 2).

**Fig. 2:**
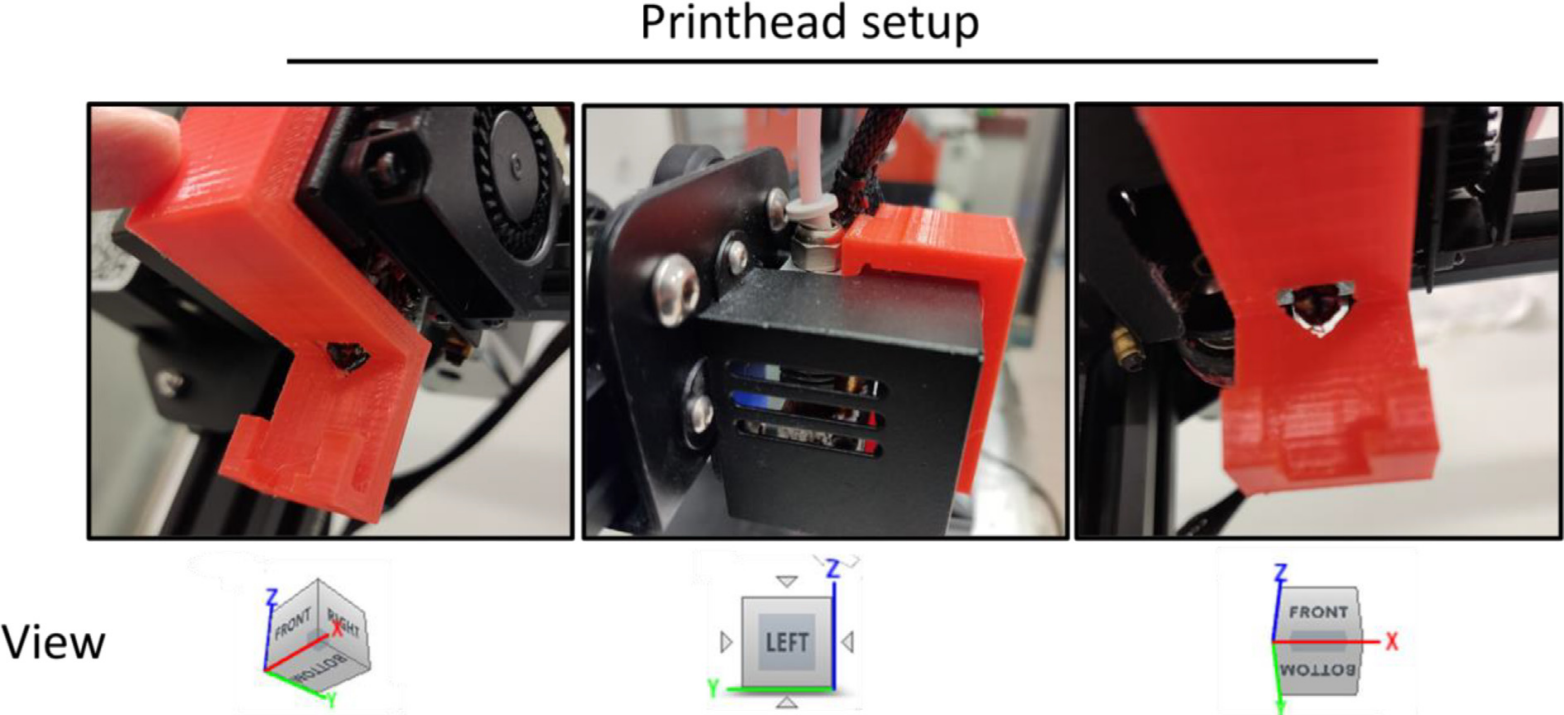
**Printhead setup.** The pictures show how the headpiece should be set up on the printhead from a bottom right angle view (left panel) a left side view (central panel) and a bottom front view (right panel). Notice how the headpiece is designed to have a lip that fits into the metal cover of the hot end, and has a small semicircular cavity that allows for a small allen wrench to be inserted to apply leverage and remove the headpiece more easily (central panel). The nozzle should align with the triangular cutout in the headpiece (right panel).
8. Test-fit the slide holder by placing it into the headpiece hook element.
9. If everything feels solidly held, proceed to the next step. Otherwise, smoothen the fit using a sharp blade or sandpaper.
10. Once everything is set, remove the headpiece and the Jar holder and follow the next paragraph to run the necessary tests before proceeding with an actual staining. It is also important to understand the g-code (available on the GitHub repository [9] and with the article) so that it can be customized to the investigator’s own needs. More details about the g-code are presented in section 6.

## Operation instructions

The HistoEnder is essentially a robot that will move the microscope slides from one staining jar to another, in a specific sequence and with precise timing. As such, after running the initial calibration, all that is left to do is to organize the jars depending on the staining or procedure that one wants to achieve, and write the gcode, i.e. the language the machine uses to direct its movements and timings. There are very few (7) g-code commands involved in this procedure:

**G90**: use absolute positioning – this command tells the Ender-3 that the g-code coordinates are relative to the machine’s actual X0 Y0 Z0 position, which the machine can easily find through its built-in end stops. This ensures procedural precision and reproducibility.

**M106 S0**: set fan speed (M106) to 0 % (S0). The procedure does not need the fan to be active so it should be off.

**M302 S0**: set extrusion temperature (M302) to 0 (S0). There is no material extrusion involved in the procedure, so there should be no heating. This would normally prevent the machine from moving, while setting it to S0 will allow any movement even when cold (formally, above 0 °C). Can be changed with **M302 *P*1** which just disables temperature checking.

**G28:** home all axes. This command moves all axes towards their end stops, until they are reached, setting, in this way, the center of the cartesian axis X0 Y0 Z0.

**G1:** linear move. This command should be followed by a set of Cartesian coordinates and optionally the feedrate (**F**) which in the case of XYZ movement, represents the speed of the movement in mm/min. Setting F to an unrealistically high value (e.g. **F5000**) will ensure that the printer will move as fast as possible, lower values (e.g. **F300**) will result in lower speeds. Not adding an F value will result in the printer moving at its default speed setting (usually as fast as possible). We used this command to limit Y speed and prevent spillage and movement of the jars onto the bed without needing to fix them to it.

**G4**: wait. This command just tells the printer to wait for a set number of seconds (**S**) or milliseconds (**P**). We use this command to set the incubation time in the jars.

**M300**: the printer beeps. This command is useful to notify the user that something specific is happening (e.g. the procedure is starting/has ended).

Additionally, semicolons (**;**) can be used after a command to add a text comment to the line, so that the reason to use that specific command in that specific line is explicit, which favours the users themselves, and others who might want to use that g-code.

Lastly, always remember that gcode accepts **one command per line**, and **do not leave blank lines** or the procedure will stop.

**Fig. 3:**
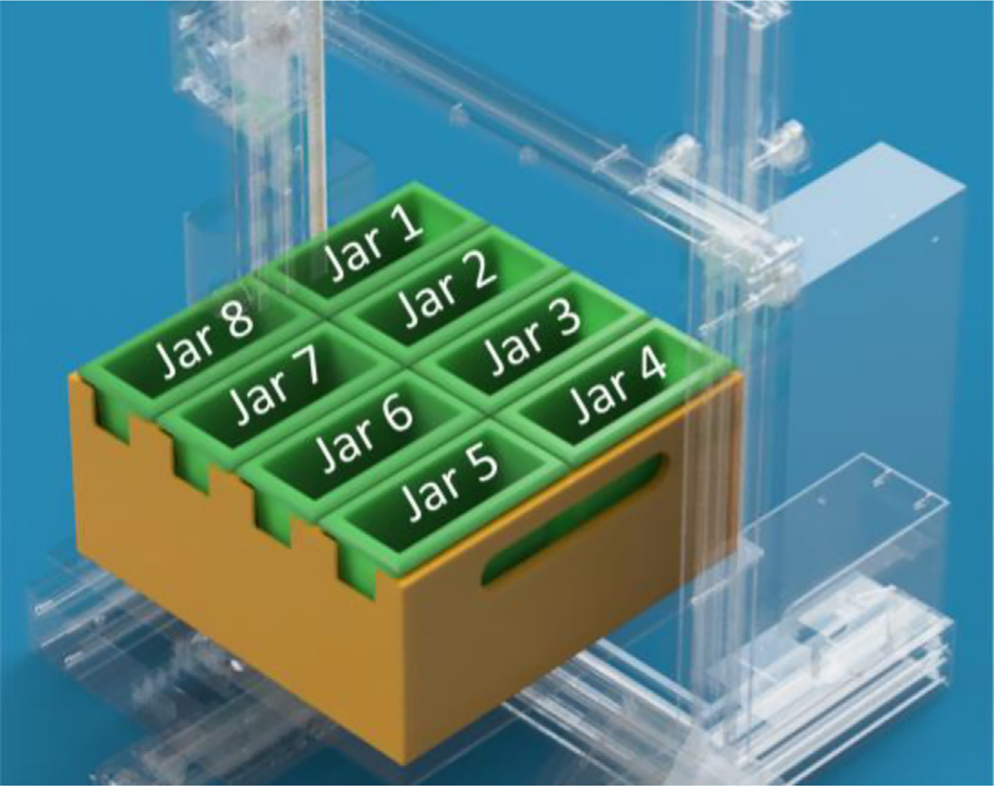
Schematic of jar system design.

At this point, all that is left to do is understand the coordinates of the jars, and the height at which slides will be free to move, and that at which they will be submerged in the solvent/staining solution. If the investigator is using our system (Fig. 3) the coordinates are specified in Table 1.

**Table 1:** XY coordinates of our jar system.

**Jar 1**	**Jar 2**	**Jar 3**	**Jar 4**
X33 Y169.5	X86.5 Y169.5	X140 Y169.5	X193.5 Y169.5

**Jar 8**	**Jar 7**	**Jar 6**	**Jar 5**
X33 Y60	X86.5 Y60	X140 Y60	X193.5 Y60

**Fig. 4:**
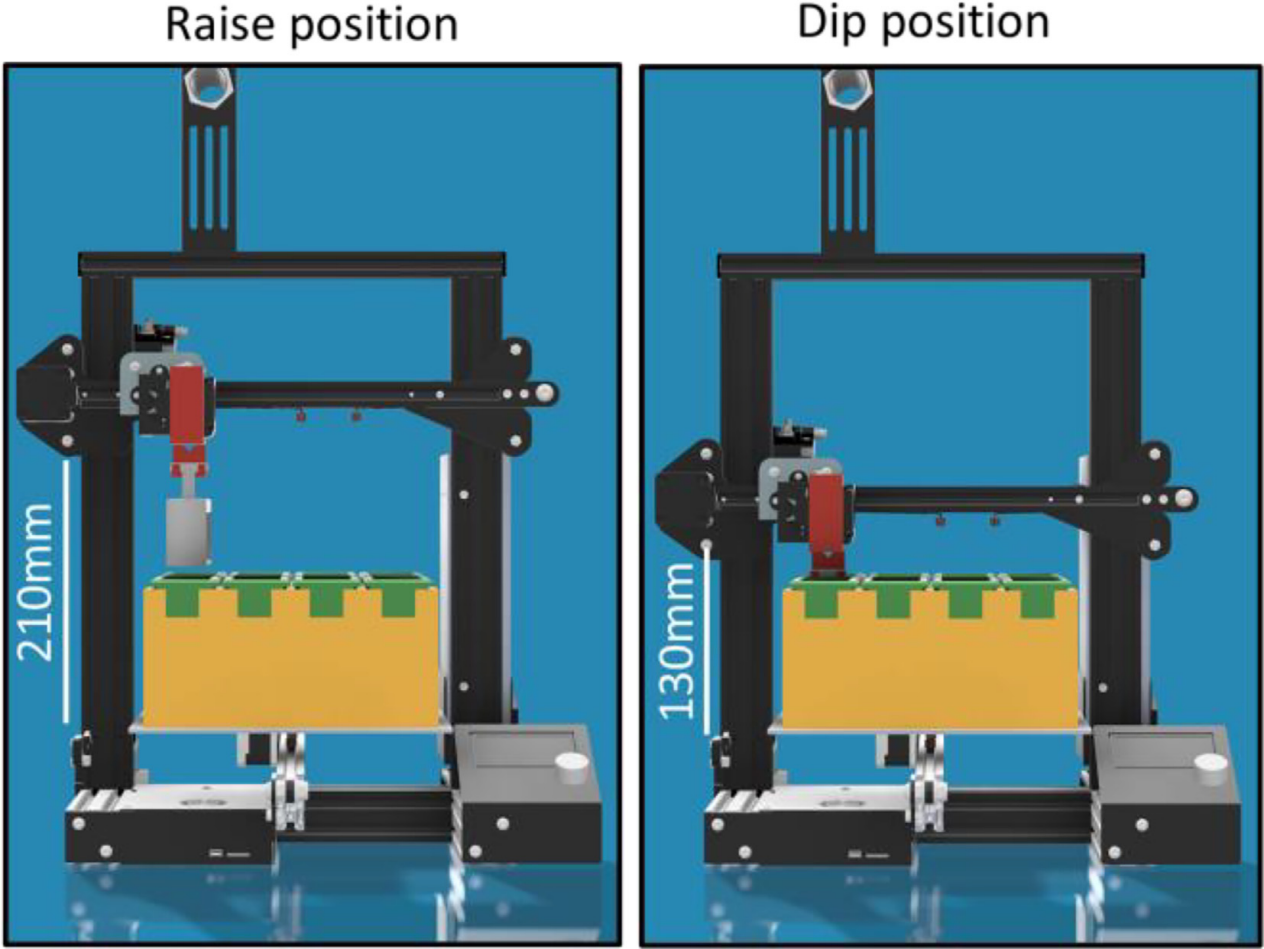
**Raise and dip positions.** 3D rendering of the HistoEnder system in its raise (Z = 210 mm) and dip (Z = 130 mm) positions.

As for Z, a height of **210** was sufficient to allow movement from one jar to the next (raise position) and a height of **130** ensured the slides were submerged in the liquid of the jar (dip position) when using the stock Ender-3 bed mat, as schematised in Fig. 4. Notice that depending on the researcher’s own setup, the numbers might need to be adjusted to be slightly higher or lower.With this knowledge, it is possible to put all the g-code commands in lines, and customize the protocol to suit different needs. It is possible to use our system, or use as many jars as one needs as long as they fit on the printer bed. And if this is not the case, it is easy and cheap enough to use one or two more jar holders with 8 more jars each, or even another HistoEnder to increase the scope of the procedures that it is possible to perform.

### Testing the system: Dry Run.gcode

Note: bracketed or bulleted parts are not to be included in the gcode.

Before starting the Dry Run.gcode program, make sure the bed sheet is taped to the bed, and the additional printed part are not yet installed on the printer.

• Start gcode

G90; use absolute positioning.

M106 S0; fan speed 0.

M302 S0; print without checking temperature.

G28; home all axes for setting X  = Y = Z = 0.

G1 Z210 F5000; move Z axis to allow installation of Jar holder and headpiece.

G1 X33 Y169.5; move X and y axis to allow placement of Jar holder and headpiece. hovers on position of Jar 1.

G4 S110; wait 110 s to allow installation of Jar holder and headpiece.

• At this point, 2 min are available to the researcher for mounting the headpiece onto the printhead, and setting the jar holder on the bed. Use the alignment guide to place the jar holder with the empty jars in it (remember to put the tabs in if needed) onto the bed. Make sure the sides of the jar holder are parallel to the sides of the bed
• hang the slide rack to the headpiece. It is advisable to balance the number of slides put in either side of the slide rack so that the rack hangs straight and does not tilt forwards or backwards. Use an empty slide if needed
• the slide rack should be hanging directly above jar 1. If this is not the case, the headpiece can be moved side to side and the Jar holder front to back to ensure proper alignment. Optionally, the investigator can mark the position on the bed and on the headpiece so that the alignment guide will not be needed in subsequent runs

M300; play tone to indicate procedure starts in 10 s.

M300.

M300.

G4 S10.

• The slide rack will now move from jar to jar until it completes the round. Keep an eye on the machine while it is doing this test run to shoot any trouble that might arise

G1 X33 Y60; move to position of Jar N°8.

G1 Z130; dip slide rack.

G4 S1; wait for n seconds of incubation in Jar.

G1 Z210 F5000; raise slide rack.

G1 X86.5 Y60; move to position of Jar N°7.

G1 Z130; dip slide rack.

G4 S1; wait for n seconds of incubation in Jar.

G1 Z210 F5000; raise slide rack.

G1 X140 Y60; move to position of Jar N°6.

G1 Z130; dip slide rack.

G4 S1; wait for n seconds of incubation in Jar.

G1 Z210 F5000; raise slide rack.

G1 X193.5 Y60; move to position of Jar N°5.

G1 Z130; dip slide rack.

G4 S1; wait for n seconds of incubation in Jar.

G1 Z210 F5000; raise slide rack.

G1 X193.5 Y169.5 F500;move to position of Jar N°4.

G1 Z130; dip slide rack.

G4 S1; wait for n seconds of incubation in Jar.

G1 Z210 F5000; raise slide rack.

G1 X140 Y169.5; move to position of Jar N°3.

G1 Z130; dip slide rack.

G4 S1; wait for n seconds of incubation in Jar.

G1 Z210 F5000; raise slide rack.

G1 X86.5 Y169.5; move to position of Jar N°2.

G1 Z130; dip slide rack.

G4 S1; wait for n seconds of incubation in Jar.

G1 Z210 F5000; raise slide rack.

G1 X33 Y169.5; move to position of Jar N°1.

G1 Z130; dip slide rack.

G4 S1; wait for n seconds of incubation in Jar.

G1 Z210 F5000; raise slide rack.

M300; play tone to indicate end of procedure.

M300.

M300.

• If everything runs smoothly, the operator is ready to mark the final positions of the jar holder and the headpiece adaptor on the tape, remove all the printed components from the assembly, fill the racks and modify the g-code to suit their needs. If there is the need to do any adjustment during the test run, start over with the new settings and repeat the dry run from the start until everything is properly set up
• Before turning off the printer, it is advisable to remove all jars and printed components from the printer bed, zero the axis, and then turn it off.
• If the researcher wants to perform an actual staining procedure right after the dry run, or wants to do another staining after finishing one, **it is IMPORTANT to remove all the printed components from the printer before starting the new g-code or modify it to avoid the re-zeroing of the axes**

## Validation and characterization

To validate the HistoEnder, we performed the most used histological staining procedure in any pathology lab, the Haematoxylin & Eosin staining starting from paraffin-embedded samples, and compared its performance to manually stained sections. Our manual protocol for this procedure requires about 1 h of hands-on time, while with the HistoEnder this time is reduced to less than 5 min.

Our Jar setup is detailed in Table 2:

**Table 2:** Contents of the Jars to perform Haematoxylin-Eosin staining using the HistoEnder.

**Jar 1**	**Jar 2**	**Jar 3**	**Jar 4**
Bioclear (Xylene substitute)	100 % Dehyol (Ethanol substitute)	95 % Dehyol in distilled water	70 % Dehyol in distilled water

**Jar 8**	**Jar 7**	**Jar 6**	**Jar 5**
1 % Eosin Y, alcohol-based	Tap water	Harris’ Haematoxylin	Distilled water

**Procedure:**

Set the paraffin-embedded sections slides into the slide rack and move the slides through the solutions as follows:

• 15 min in Bioclear (jar 1)
• 4 min in 100 % Dehyol (jar 2)
• 4 min in 95 % Dehyol (jar 3)
• 4 min in 70 % Dehyol (jar 4)
• 4 min in Distilled water (jar 5)
• 25 s in Harris’ Haematoxylin (jar 6)
• 5 min in tap water (jar 7)
• 45 s in Eosin Y (jar 8)
• 2 min in 70 % Dehyol (jar 4)
• 2 min in 95 % Dehyol (jar 3)
• 2 min in 100 % Dehyol (jar 2)
• 10 min in Bioclear (jar 1)

then mount the slides in a xylene-based medium (e.g. Eukitt, Sigma-Aldrich) and observe with a brightfield microscope.

**Fig. 5:**
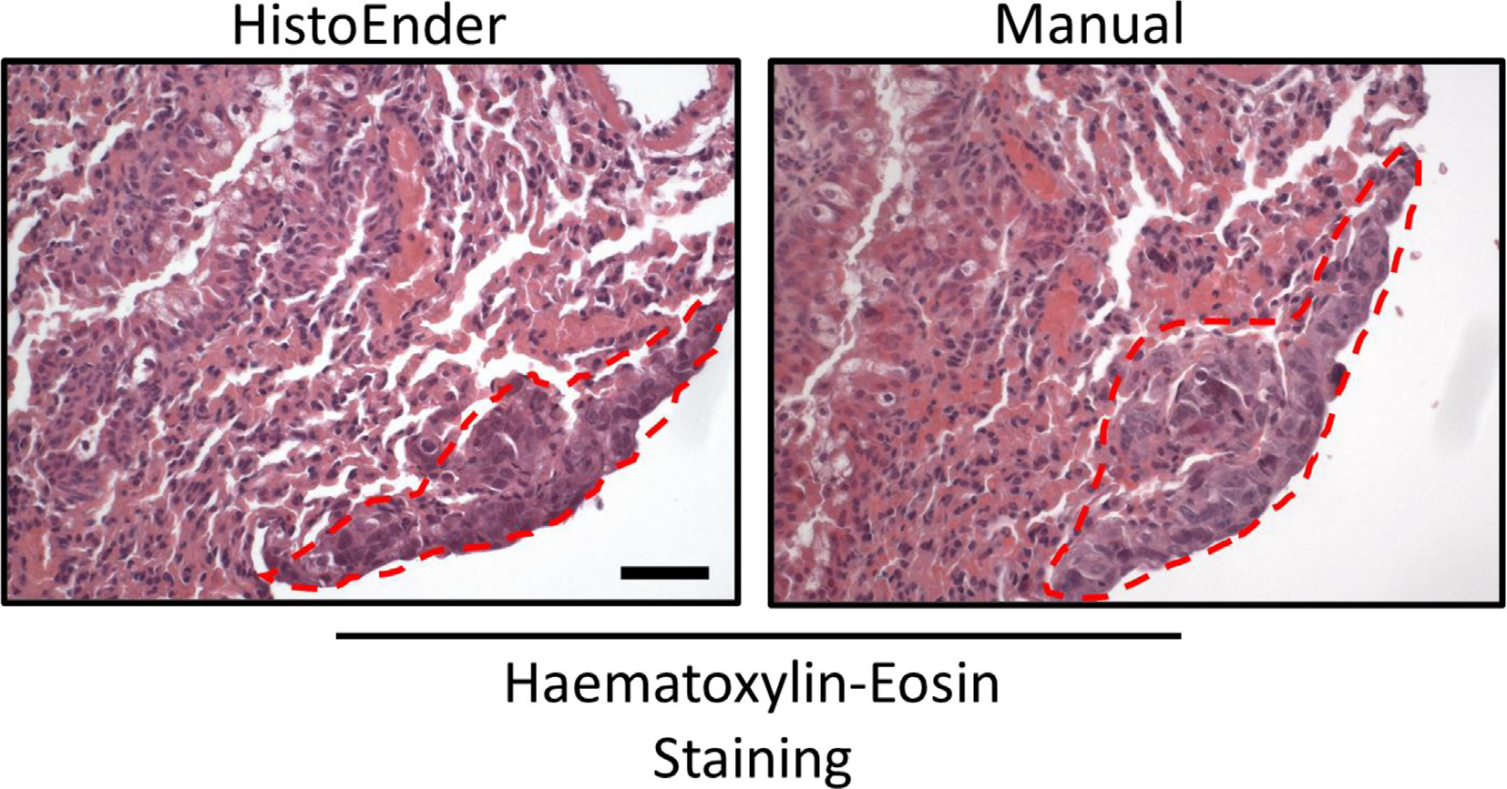
**Haematoxylin-eosin staining – HistoEnder *vs* manual staining.** Lungs arising from an archival *in vivo* experiment were fixed in paraformaldehyde, embedded in paraffin, sectioned in 5 µm-thick serial sections and processed for haematoxylin & eosin staining, either with the HistoEnder (left panel) or manually (right panel). The sections chosen for the staining were serial. Red dotted line marks an osteosarcoma lung metastasis. Bar = 50 µm. (For interpretation of the references to colour in this figure legend, the reader is referred to the web version of this article.)

This procedure was quite easy to translate into gcode (see HaematoxylinEosin.gcode), and the staining results were indistinguishable between manually stained and HistoEnder stained sections (Fig. 5). In this case, the staining allowed for the identification of an osteosarcoma lung metastatic focus.

There are countless more stainings that are possible to do with a HistoEnder, including some very important pathology-related ones such as the standard Papanicolaou stain used in pap tests [2], the picrosirius red stain to detect fibrotic tissue [15], periodic acid-Shiff stain to detect glycogen and similar molecules [16], alcian blue to detect acid mucins that are accumulated in mesotheliomas [17], and more. Even when the staining is too complicated to be easily done by a machine, as is the case for immunohistochemistry [18], *in situ* hybridisation [19], or tartrate-resistant acid phosphatase (TRAcP) staining [20] the HistoEnder can still be of assistance, for example by saving the time needed to manually hydrate the slides, or by performing washes, that can be tedious and time consuming especially with multiple slides. For example, in a recent report [21], we used the HistoEnder to perform toluidine blue staining, and the initial phases of the TRAcP staining on PMMA-embedded bone samples, which proved themselves to be indistinguishable from the manually stained ones.

It is also important to underline the limitations of the machine. The main one is that the number of jars is limited to 8 in this setup. One could use more than 8 jars, but to fit them onto the Ender-3 bed, they would need to be smaller and therefore less slides could be processed in a single run. A simple solution would be to print two or more jar holders, load them up with jars containing different solvents or stains, introduce a short pause in the g-code to allow switching the two jar holders, and then go on with the procedure. Another option would be using two or more HistoEnders in tandem, which would still be affordable given the value such a system provides. Moreover, the system described here is adaptable to any FDM printer. An FDM printer with a larger printing bed, for example 40x40 cm would allow the use of more jars at the same time. Another limitation is that with the current design of the headpiece, it may not be possible to use all 25 slide slots on the slide holder. We were able to use up to 23 maximum. An easy workaround would be drilling holes in the slide rack and using those to mount it to a re-designed headpiece, however this would make it more difficult to do for some labs therefore we decided not to include this procedure here. In the future, we aim to expand the scope of the HistoEnder and to make it easier to use by creating a specific software for g-code generation, connecting the printer wirelessly, and by controlling it remotely. Having linear rails and belts to move the Z axis is also a modification we are considering since this would allow the movement to be faster, providing a more precise staining time for very quick-acting dyes, e.g. Gill’s Haematoxylin #3 which can require times as short as 5 s to provide adequate staining.

**Ethics statements**.

The sections used for the stainings arose from an *in vivo* experiment carried out on rodents, that was carried out in accordance with the U.K. Animals (Scientific Procedures) Act, 1986 and associated guidelines; EU Directive 2010/63/EU for animal experiments; or the National Institutes of Health guide for the care and use of laboratory animals (NIH Publications No. 8023, revised 1978). Mice were 7-week-old (at sacrifice) balb/c female nude animals inoculated partially with MNNG/HOS osteosarcoma cells.

## Declaration of Competing Interest

The authors declare that they have no known competing financial interests or personal relationships that could have appeared to influence the work reported in this paper.
